# Lack of a Y-Chromosomal Complement in the Majority of Gestational Trophoblastic Neoplasms

**DOI:** 10.1155/2010/364508

**Published:** 2010-02-21

**Authors:** Kai Lee Yap, Michael J. Hafez, Tsui-Lien Mao, Robert J. Kurman, Kathleen M. Murphy, Ie-Ming Shih

**Affiliations:** ^1^Department of Pathology, Johns Hopkins Medical Institutions, Baltimore, MD 21231, USA; ^2^Pathobiology Graduate Program, Johns Hopkins University School of Medicine, Baltimore, MD 21231, USA; ^3^Department of Pathology, National Taiwan University Hospital, Taipei 100, Taiwan; ^4^Department of Gynecology, Obstetrics and Oncology, Johns Hopkins Medical Institutions, Baltimore, MD 21231, USA

## Abstract

Gestational trophoblastic neoplasms (GTNs) are a rare group of neoplastic diseases composed of choriocarcinomas, placental site trophoblastic tumors (PSTTs) and epithelioid trophoblastic tumors (ETTs). Since these tumors are derivatives of fetal trophoblastic tissue, approximately 50% of GTN cases are expected to originate from a male conceptus and carry a Y-chromosomal complement according to a balanced sex ratio. To investigate this hypothesis, we carried out a comprehensive analysis by genotyping a relatively large sample size of 51 GTN cases using three independent sex chromosome genetic markers; Amelogenin, Protein Kinase and Zinc Finger have X and Y homologues that are distinguishable by their PCR product size. We found that all cases contained the X-chromosomal complement while only five (10%) of 51 tumors harbored the Y-chromosomal complement. Specifically, Y-chromosomal signals were detected in one (5%) of 19 choriocarcinomas, one (7%) of 15 PSTTs and three (18%) of 17 ETTs. The histopathological features of those with a Y-chromosome were similar to those without. Our results demonstrate the presence of a Y-chromosomal complement in GTNs, albeit a low 10% of cases. This shortfall of Y-chromosomal complements in GTNs may reinforce the notion that the majority of GTNs are derived from previous molar gestations.

## 1. Introduction

Gestational trophoblastic neoplasms (GTNs) represent a unique group of human tumors that develop as a semiallograft from fetus-derived tissues [1]. GTNs were originally considered a homogeneous group of diseases arising from the neoplastic transformation of trophoblastic cells. However, recent clinicopathological studies have provided evidence that there are at least three distinctive types of GTNs including the most common type, choriocarcinomas, and the less common placental site trophoblastic tumors (PSTTs) and epithelioid trophoblastic tumors (ETTs). GTNs have been proposed to develop from trophoblastic stem cells, presumably the cytotrophoblastic cells, and the patterns of differentiation in GTNs recapitulate the early stages of placental development [1]. According to this view, choriocarcinomas are composed of variable amounts of neoplastic cytotrophoblasts, syncytiotrophoblasts, and extravillous (intermediate) trophoblasts, resembling the previllous blastocyst which is composed of a similar mixture of trophoblastic subpopulations. In contrast, the neoplastic cytotrophoblasts in PSTTs differentiate mainly into extravillous (intermediate) trophoblastic cells; whereas the neoplastic cytotrophoblasts in ETTs differentiates into chorionic-type extravillous (intermediate) trophoblastic cells. According to this model, choriocarcinomas are the most primitive trophoblastic tumors, whereas PSTTs and ETTs are relatively more differentiated. 

Clinically, choriocarcinoma is a highly malignant epithelial tumor arising from the trophoblasts of any type of gestational event, most often a complete hydatidiform mole [2]. Patients are in their reproductive age and present with abnormal vaginal bleeding and occasionally signs of distant metastasis. Microscopically, it predominantly consists of a biphasic proliferation of mononucleate trophoblasts and syncytiotrophoblasts, accompanied by prominent hemorrhage and necrosis [3]. With the advent of modern chemotherapy, the overall survival for patients with choriocarcinomas currently approaches 100% [4] although some patients develop nonoperable and chemoresistant recurrent disease. As compared to choriocarcinomas, PSTTs and ETTs are rare [5–7]. Like choriocarcinomas, PSTTs and ETTs occur in women of reproductive age and present with either amenorrhea or abnormal bleeding [5, 7, 8]. Despite deep myometrial invasion, most cases of PSTTs and ETTs are successfully treated [2] but approximately 10–15% are clinically malignant and have a fatal outcome. Histologically, PSTTs are characterized by masses or sheets of intermediate trophoblastic cells resembling implantation site intermediate trophoblasts, while ETTs are characterized by chorionic-type intermediate trophoblasts of the normal implantation site and placenta. In addition to distinct morphological features, both PSTTs and ETTs are characterized by unique gene expression patterns, suggesting that the molecular pathogeneses of PSTTs and ETTs are dissimilar [9]. 

Hydatidiform moles [10, 11] are precursor lesions of numerous cases of GTNs. Previous clinicopathological and molecular studies have provided fundamental insight into the pathogenesis of hydatidiform moles but the molecular and cellular basis for the development of GTNs remain poorly understood. A similar number of GTN cases with and without a Y-chromosome are expected if sex chromosomes play no role in the development of GTNs. On the contrary, more than 85% of patients with PSTTs were found by history records or genetic analysis to have had a female antecedent gestation. Moreover, a recent study using the Amelogenin assay demonstrated the presence of a X-chromosome and absence of a Y in a small series of PSTTs [12], raising the possibility that a Y-chromosomal complement may be preferentially deleted in PSTTs. In this paper we describe our findings in a larger number of PSTTs, as well as other types of GTNs including choriocarcinomas and ETTs. In addition, unlike previous studies using a single marker, we examined a total of three genetic markers including the commonly used amelogenin. These genes have X and Y homologues that can be distinguished by their polymerase chain reaction (PCR) product sizes using specific primer pairs, to detect the presence of a Y allele. 

## 2. Methods

### 2.1. Tissue Specimens

Paraffin tissues from a total of 51 GTNs were retrieved from the archival files in the Department of Pathology at the Johns Hopkins Hospital, USA. Most of the specimens were consultation cases sent to two of the authors (R. J. Kurman and I. M. Shih). Hematoxylin and eosin stained sections from tissue specimens were reviewed and the diagnosis of specific types of GTNs were confirmed by an expert gynecologic pathologist (I. M. Shih). The specimens included 15 PSTTs, 17 ETTs, and 19 choriocarcinomas. All the specimens were anonymized and thus clinical information was not obtained. Tissues collection was conducted in compliance with institutional review board regulations. The tumor areas on paraffin sections were carefully dissected from the surrounding normal (maternal) tissues on hematoxylin-stained tissue sections. Genomic DNA was prepared by using the Formapure kit (Agencourt, Cambridge, MA). One representative tissue block was selected for DNA extraction except for five cases in which the DNA was purified from two separate tissue blocks.

### 2.2. Genotyping Using Sex Chromosome-Specific Genetic Markers

The presence of either a X or a Y-chromosome in GTNs was determined by the analysis of three genes that have X and Y-chromosomal homologues distinguishable by their PCR product size with specific primer sets; *Amelogenin* X and Y (AMELX and AMELY), *Protein Kinase* X and Y (*PRKX* and *PRKY*), and *Zinc Finger *X and Y (*ZFX* and *ZFY*). The amelogenin gene has X and Y homologues located on Xp22.1–22.3 (*AMELX*) and Yp11.2 (*AMELY*), which are differentiated using a primer pair that amplifies a region of intron 1 which spans a 6-base pair deletion in *AMELX* as compared to *AMELY*. The Amelogenin analysis was performed using the commercially available AmpFlSTR Profiler kit (Applied Biosystems, Foster City, CA). Thermal cycling conditions and capillary electrophoresis were carried out according to the manufacturer's instructions. Briefly, the PCR conditions were 95°C for 11 minutes followed by 28 cycles of 94°C for 1 minute, 59°C for 1 minute, and 72°C for 1 minute, followed by a final extension at 60°C for 45 minutes. After amplification, capillary electrophoresis was carried out using 1 *μ*l of multiplex PCR product, mixed with 9 *μ*l of deionized formamide/GeneScan 500 (ROX) size standard (Applied Biosystems). Samples were then denatured at 95°C for 2 minutes before analysis on the ABI3100 Genetic Analyzer (Applied Biosystems).

Although the Amelogenin-based sex chromosome assay has been frequently used in basic research and forensic medicine, a false negative result for the detection of a Y-chromosome has been documented when tumors of high genomic instability are analyzed [13]. Therefore, we analyzed two additional genes with X and Y homologues. The *PRK* gene has its X and Y homologues located on Xp22.3 (*PRKX*) and Yp11.2 (*PRKY*) respectively. The *PRKY* gene is located approximately 0.35 Mb centromeric to *AMELY*. To differentiate *PRKX* and *PRKY*, we designed a PCR reaction to amplify exon 8 of the *PRKX* and *PRKY* genes, using a primer set that spans a three-base pair deletion (Table 1) [14]. The *PRKY* amplification product is three bases shorter than the *PRKX* product. The *ZF* gene has X and Y homologues located at Xp22.1 (*ZFX*) and Yp11.2 (*ZFY*), respectively. *ZFY* is located approximately 3.9 Mb telomeric to *AMELY*. To differentiate *ZFX* and *ZFY*, we designed a PCR reaction to amplify exon 3 of the *ZFX* and *ZFY* genes, with the primer set also spanning a 3-base pair deletion. In this case, the *ZFX* product is 3 bases shorter than the *ZFY* product. PCR amplifications were carried out and the primer sequences were listed in Table 1. Reactions were thermal cycled using the touchdown protocol: 1 cycle of 95°C for 2 minutes, 3 cycles each of 94°C for 30 seconds, 64°C, 61°C, or 58°C for 30 seconds, and 70°C for 30 seconds. This was followed by 35 cycles of 94°C for 30 seconds, 57°C for 30 seconds and 70°C for 30 seconds, and 1 cycle of 70°C for 5 minutes. Products were analyzed by capillary electrophoresis as described above.

## 3. Results and Discussion

**Figure 1:**
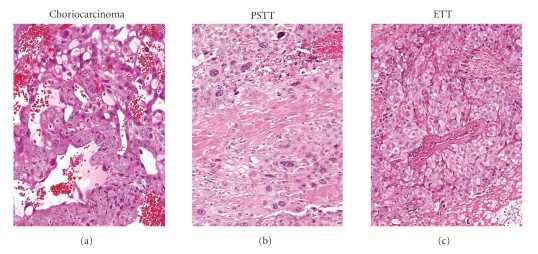
Histological features of gestational trophoblastic neoplasms. Choriocarcinoma is characterized by biphasic growth pattern composed of syncytiotrophoblast and mononucleate trophoblastic cells, forming vasculogenic mimicry. Placental site trophoblastic tumor (PSTT) is composed of confluent masses of neoplastic intermediate (extravillous) trophoblastic cells, infiltrating within smooth muscle cells. Epithelioid trophoblastic tumor (ETT) contains neoplastic chorionic-type intermediate (extravillous) trophoblastic cells surrounding an artery.

**Figure 2:**
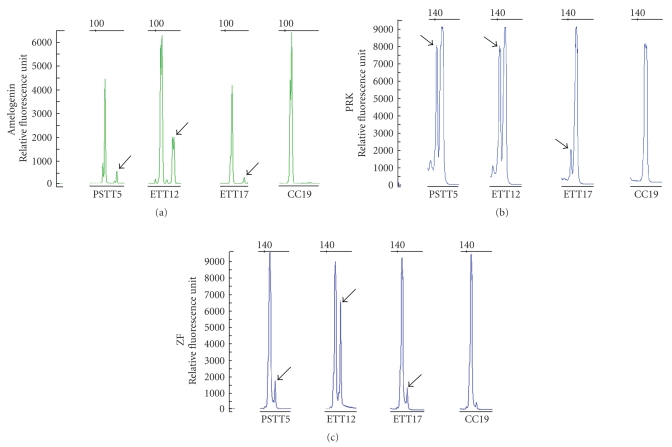
Genotypes in representative trophoblastic tumor specimens. The presence of either the X or Y-chromosome in GTNs was determined by the analysis of three genes that have X and Y homologues distinguishable by their PCR product size; *Amelogenin* X and Y (*AMELX* and *AMELY*), *Protein Kinase* X and Y (*PRKX* and *PRKY*), and *Zinc Finger *X and Y (*ZFX* and *ZFY*). Arrows denote the Y chromosomal peaks.

A total of 51 GTNs were histologically reviewed, including 19 choriocarcinomas, 17 ETTs, and 15 PSTTs (Table 2). Of these samples, ETT17 contained a small area of choriocarcinoma and PSTT15 contained a focal ETT component. Representative histologic features of the GTNs are illustrated in Figure 1. All cases yielded informative results in at least one of the gene markers utilized for the sex chromosome genotyping. We found that all informative cases contained a X-chromosomal complement while only five (10%, 95% CI: 18.2%–1.8%) of 51 tumors harbored a Y chromosome complement (Table 2). Specifically, Y-chromosomal signals were detected in one (5%) of 19 choriocarcinomas, one (7%) of 15 PSTTs, and three (18%) of 17 ETTs (Table 2). Figure 2 illustrates the genotypes in representative specimens. Of note, the genotypes were identical in genomic DNA obtained from different tissue blocks of the same case. For those specimens with a Y-chromosome, all three gene markers revealed consistent outcomes, although the relative abundance of a Y gene versus an X gene varied. For example, PSTT5 showed a small Y peak in both *amelogenin* and *ZF* loci that could make the Y assignment equivocal (Figure 2). However, by analyzing *PRKY*, we clearly detected a robust *PRKY* peak from the same specimen. Similarly, ETT12 contained a relatively small amelogenin Y peak but had significantly large peaks at both *PRKY* and *ZFY*. These findings indicate the variable efficiency of primers that amplify the different Y loci of the three genes on formalin-fixed paraffin tissues and underscore the importance to include additional markers to assess the presence of Y-chromosomal elements. The histopathological features in those tumors with a Y-chromosome were indistinguishable from those without a Y-chromosome. The percentage of cases showing Y peaks is listed in Table 3. 

The lack of a Y-chromosomal complement in the majority of GTNs is intriguing and several theories can account for this phenomenon. The most likely cause of the phenomenon is that Y-chromosomal deletions have no functional effects on tumor progression [15]. In this case, the absence of Y chromosome in GTNs may simply reflect the fact that many GTNs develop from complete hydatidiform moles of which approximately 90% contain a karyotype of 46,XX due to fertilization of an “empty” ovum (without nucleus) by a single haploid (23X) sperm followed by haploid genome duplication [10, 11]. Thus, the GTNs that develop from complete hydatidiform moles retain the same sex chromosome assignment as their precursors and do not harbor a Y-chromosome. While 90% of complete hydatidiform moles arise from monospermy, approximately 10% are due to fertilization of an empty ovum with two sperm. Half of these cases that arise from dispermy would be expected to carry a Y-chromosome. Thus it could be predicted that approximately 5% of complete hydatidiform moles, and their resulting choriocarcinomas, would carry a Y-chromosome, which is exactly the percentage we obtained in this study.

Although the above represents our favorite view, other interpretations should also be indicated. It is possible that Y-chromosome deletions have a functional implication in the development of GTNs. In addition to GTNs developing from trophoblastic cells of a female conceptus, it can be speculated that GTNs arising from trophoblastic cells of a male conceptus will undergo clonal selection of trophoblastic cells with a deleted Y-chromosome due to their underlying genomic instability. In both scenarios, it is assumed that the presence of a Y-chromosome is not compatible with tumor initiation, possibly due to potential growth-inhibitory effects conferred by the products of genes located in the Y-chromosome. In support of this notion is the observation of a small but unambiguous Y peak of *AMELY*, *PRKY* and *ZFY *in the carefully dissected ETT17 (Figure 2). Also, previous reports have demonstrated Y-chromosome loss in several types of human cancer including prostate carcinoma, renal cell carcinoma, acute promyelocytic leukemia, and head and neck squamous carcinoma [15–18]. 

Lastly, the lack of Y-chromosome detection in other studies may be the result of micro-deletions in the Y-chromosomal regions analyzed, yielding a false negative result. This technical pitfall has been well documented in solid tumors when the amelogenin-based assay was applied [13]. To overcome this problem, in this study we have included two additional gene markers, *PRK* and *ZF*, along with the standard amelogenin test. Similar to the *Amelogenin (AMEL)* locus, the *PRK,* and *ZF* genes have X and Y homologues located on Xp (*PRKX* and *ZFX*) and Yp (*ZFX* and *ZFY*). The *PRKY *and *ZFY* are located 3.9 Mb telomeric to *AMELY* and 0.35 Mb centromeric to *AMELY, *respectively. The failure to detect any of the three genes of the Y chromosome derives a more definitive conclusion and suggests that the absence of Y-chromosome is not likely due to somatic micro-deletions or microsatellite instability of the Y-chromosome-associated loci in GTNs.

Among 51 GTNs analyzed, we detected Y alleles in five tumors based on the presence of Y peaks in at least one of the *AMELY*, *PRKY* and *ZFY* loci. Among these five tumors was a PSTT. This finding is in contrast to a previous report demonstrating that none of 13 PSTTs harbored the *AMELY* [12]. The discrepancy is likely explained by the larger sample size and the additional Y markers employed in this study. The conclusion from the current study is also different from our previous report showing that approximately half of PSTTs and ETTs contained the sex-determining region Y (SRY) on Y chromosome [19]. In that study, a high cycle number of PCR amplification was used in order to detect a limited source of genomic DNA from paraffin tissues, raising the possibility of nonspecific amplification from contaminants. Thus, we believe that the results from the current study are more definitive in determining the sex chromosome assignment of GTNs. 

In conclusion, this study provides a comprehensive analysis of sex chromosome distributions in all types of GTNs using three independent gene markers with differing PCR product lengths in the X and Y-chromosomes when specific primer pairs are used. Our results, based on a relatively large number of cases, clearly demonstrate the presence of a distinct but low Y-chromosomal complement in choriocarcinomas, PSTTs, and ETTs, that contributes to an overall figure of approximately 10%. It is most likely that the shortfall of Y chromosomal complements in GTNs may simply be due to the genetic basis of their precursor lesions, complete hydatidiform moles in which the majority of cases had the genotype of XX [20]. In conclusion, our results suggest that the majority of GTNs are preceded by antecedent complete molar pregnancy, many of which may be under recognized as the early complete moles usually lack the characteristic histopathological features. 

## Figures and Tables

**Table 1:** The primer sequences used to amplify the *PRK* and *ZF* loci.

Primer Name	Sequence
PRK-F	5′ FAM-TTTTGTTTCTTTCTGTCCATACTTAAAG 3′
PRK-R	5′ TCCCAAACCACTCAACTG 3′
ZF-F	5′ FAM-TGTGCATAACTTTGTTCCTGATG 3′
ZF-R	5′ AGCACTTGCTCAGGAATGATG 3′

**Table 2:** The sex chromosome assignment in all the GTN samples.

Case	Diagnosis	AME	PRK	ZF	Y peak
CC1	CC	XX	XX	XX	no
CC2	CC	XX	NA	XX	no
CC3	CC	XX	XX	XX	no
CC4	CC	XX	XX	XX	no
CC5	CC	XX	NA	XX	no
CC6	CC	NA	NA	XX	no
CC7	CC	XX	NA	NA	no
CC8	CC	XX	NA	XX	no
CC9	CC	XX	XX	XX	no
CC10	CC	XX	XX	XX	no
CC11	CC	XY	NA	NA	**yes **
CC12	CC	XX	XX	XX	no
CC13	CC	XX	XX	XX	no
CC14	CC	NA	NA	XX	no
CC15	CC	NA	XX	NA	no
CC16	CC	XX	NA	XX	no
CC17	CC	XX	XX	XX	no
CC18	CC	XX	XX	NA	no
CC19	CC	XX	XX	XX	no
ETT1	ETT	XX	XX	XX	no
ETT2	ETT	XX	XX	XX	no
ETT3	ETT	XX	NA	XX	no
ETT4	ETT	XX	XX	XX	no
ETT5	ETT	XX	XX	XX	no
ETT6	ETT	XX	XX	XX	no
ETT7	ETT	NA	XX	XX	no
ETT8	ETT	XX	XX	XX	no
ETT9	ETT	XX	XX	XX	no
ETT10	ETT	XX	XX	XX	no
ETT11	ETT	XX	XX	NA	no
ETT12	ETT	XY*	XY	XY	**yes **
ETT13	ETT	XX	XX	XX	no
ETT14	ETT	XY	XY	XY	**yes **
ETT15	ETT	XX	NA	XX	no
ETT16	ETT	XX	XX	XX	no
ETT17	ETT + CC	XY*	XY*	XY*	**yes **
PSTT1	PSTT	XX	XX	XX	no
PSTT2	PSTT	NA	NA	XX	no
PSTT3	PSTT	XX	XX	XX	no
PSTT4	PSTT	XX	XX	XX	no
PSTT5	PSTT	XY*	XY	XY*	**yes **
PSTT6	PSTT	NA	NA	XX	no
PSTT7	PSTT	NA	XX	XX	no
PSTT8	PSTT	XX	NA	XX	no
PSTT9	PSTT	XX	NA	XX	no
PSTT10	PSTT	NA	XX	XX	no
PSTT11	PSTT	NA	NA	XX	no
PSTT12	PSTT	XX	XX	XX	no
PSTT13	PSTT	XX	XX	NA	no
PSTT14	PSTT	XX	XX	XX	no
PSTT15	PSTT + ETT	XX	XX	XX	no

CC: CHORIOCARCINOMA, ETT: EPITHELIOID TROPHOBLASTIC TUMOR, PSTT: PLACENTAL SITE TROPHOBLASTIC TUMOR.

**Table 3:** Summary of percentage of tumor cases positive for a Y allele for at least one marker.

	Choriocarcinoma	ETT	PSTT
Total case no.	19	17	15
With Y peaks	1	3	1
% with Y peaks	5.3%	17.6%	6.7%
CI (95%)	15.4%–0%	35.7%–0%	19.4%–0%

ETT: epithelioid trophoblastic tumor; PSTT: placental site trophoblastic tumor; CI: confidence interval.
